# Machine Learning-Empowered FTIR Spectroscopy Serum Analysis Stratifies Healthy, Allergic, and SIT-Treated Mice and Humans

**DOI:** 10.3390/biom10071058

**Published:** 2020-07-16

**Authors:** Elke Korb, Murat Bağcıoğlu, Erika Garner-Spitzer, Ursula Wiedermann, Monika Ehling-Schulz, Irma Schabussova

**Affiliations:** 1 Institute of Specific Prophylaxis and Tropical Medicine, Medical University of Vienna, 1090 Vienna, Austria; elke.korb@meduniwien.ac.at (E.K.); erika.garner-spitzer@meduniwien.ac.at (E.G.-S.); ursula.wiedermann-schmidt@meduniwien.ac.at (U.W.); 2 Institute of Microbiology, Department of Pathobiology, University of Veterinary Medicine, 1210 Vienna, Austria; muratbagci2@gmail.com

**Keywords:** FTIR spectroscopy, allergy, specific immunotherapy, allergic airway inflammation, serum, clinical diagnostics, metabolic fingerprinting, deep learning, convolutional neural networks, machine learning

## Abstract

The unabated global increase of allergic patients leads to an unmet need for rapid and inexpensive tools for the diagnosis of allergies and for monitoring the outcome of allergen-specific immunotherapy (SIT). In this proof-of-concept study, we investigated the potential of Fourier-Transform Infrared (FTIR) spectroscopy, a high-resolution and cost-efficient biophotonic method with high throughput capacities, to detect characteristic alterations in serum samples of healthy, allergic, and SIT-treated mice and humans. To this end, we used experimental models of ovalbumin (OVA)-induced allergic airway inflammation and allergen-specific tolerance induction in BALB/c mice. Serum collected before and at the end of the experiment was subjected to FTIR spectroscopy. As shown by our study, FTIR spectroscopy, combined with deep learning, can discriminate serum from healthy, allergic, and tolerized mice, which correlated with immunological data. Furthermore, to test the suitability of this biophotonic method for clinical diagnostics, serum samples from human patients were analyzed by FTIR spectroscopy. In line with the results from the mouse models, machine learning-assisted FTIR spectroscopy allowed to discriminate sera obtained from healthy, allergic, and SIT-treated humans, thereby demonstrating its potential for rapid diagnosis of allergy and clinical therapeutic monitoring of allergic patients.

## 1. Introduction

Increasing numbers of allergic patients worldwide emphasize the need for improvement of diagnosis and therapy. Allergen-specific immunotherapy (SIT) is an effective and widely used treatment strategy [1]. Successful SIT leads to reduced levels of allergen-specific IgE, while specific IgG1, IgG4, and IgA are increased [1]. Hence, the determination of specific IgE levels in serum using ELISA or microarrays is used to diagnose allergies and to monitor the effectiveness of SIT [2]. Furthermore, SIT is associated with reduced Th2 cytokines IL-4 and IL-5 and increased IL-10, TGFβ, and IFNγ levels and expanded CD4^+^CD25^+^ regulatory T cells [3]. Similarly, as in humans, induction of tolerance in mice also leads to altered allergen-specific responses [4]. Interestingly, metabolite profiling of serum with nuclear magnetic resonance revealed distinct metabolic patterns between asthmatic patients and healthy controls, such as dissimilar levels of formate, acetate, choline, glutamine, and methionine [5]. Therefore, besides antibodies and cytokines, metabolic alteration profiles in serum may serve as a potential surrogate for allergy diagnostic or predictive markers of SIT efficacy.

Machine learning-assisted Fourier Transform Infrared (FTIR) spectroscopy is a rapid, reproducible, and robust approach based on the characterization of the molecular structure and conformation of biomolecules, providing spectral fingerprints from biological specimens [6]. As of its high-resolution power, easy sample preparation, and low running costs, FTIR spectroscopy might represent an interesting and economic alternative for clinical routine diagnostics. For instance, FTIR spectroscopy was used to perform metabolic analysis of rat bronchoalveolar lavage fluid (BALF) and is a suitable tool for bacterial subtyping, based on spectral metabolic fingerprints [7,8,9,10]. The rapid developments in machine-based learning also open new fields of application of FTIR spectroscopy in biology and biomedicine [11,12,13]. For instance, recently FTIR spectroscopy was adopted to discriminate between serum from cancer patients and healthy controls [14,15]. This pilot study aimed to investigate whether machine learning-powered FTIR spectroscopy could be applied as a rapid diagnostic tool to detect metabolite alterations in the serum of healthy versus allergic mice and humans for diagnosis of allergy. Thus, we used healthy, allergic, and tolerized mice, which exhibit distinct immunological phenotypes, as a model system. Furthermore, we recruited allergic and SIT-treated patients as well as healthy controls to evaluate the feasibility of FTIR measurements in human serum samples.

## 2. Material and Methods 

### 2.1. Mice

Female, 5–6 weeks old wild type BALB/c mice were purchased from Charles River. Mice were kept under conventional housing conditions. Two independent experiments, with *n* = 5 per treatment group and experiment, were carried out. Experiments were approved by the Ethics Committee of the Medical University of Vienna and the Austrian Federal Ministry of Education, Science, and Research (BMWF-66.009/0384-WFW/V/3b/2015).

### 2.2. Experimental Design and Measurement of Airway Hyperresponsiveness 

**Figure 1:**
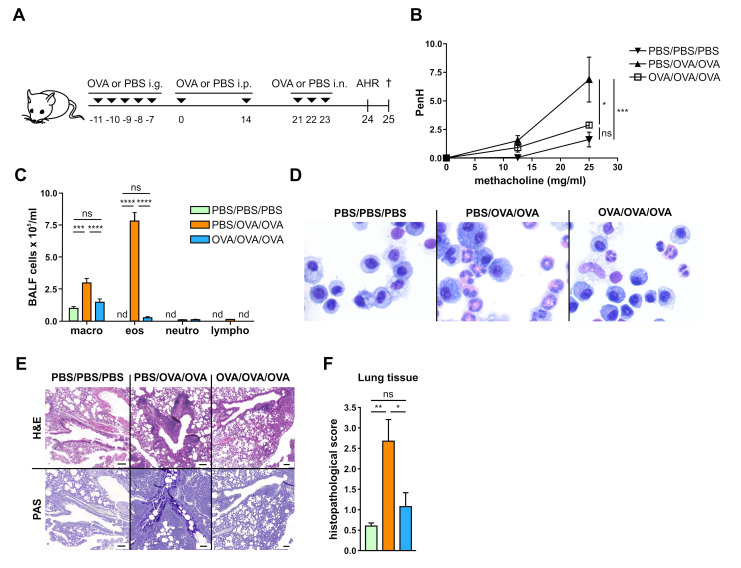
Prophylactic oral treatment with ovalbumin (OVA) leads to the induction of tolerance. (**A**), Experimental design. i.g., intragastric; i.p., intraperitoneal; i.n., intranasal; PBS, phosphate-buffered saline. (**B**), airway hyperresponsiveness (AHR) in response to methacholine. PenH, enhanced pause. (**C**), Differential cell counts in bronchoalveolar lavage fluid (BALF). (**D**), representative hematoxylin and eosin (H&E)-stained cytospins from BALF. (**E**), H&E and Periodic Acid Schiff (PAS)-stained lung sections from 1 representative example from each group. (**F**), pooled histopathological score of lung tissue. Graphs show results from 1 representative experiment with 5 mice per group (**B**) or pooled results from 2 independent experiments with 9–10 mice per group (**C**) or 1 representative example with 5 mice (**D**) or 8 mice (**E**) per group or pooled results from 2 independent experiments with 8 mice per group (**F**). Error bars show mean ± SEM. nd not detectable; ns not significant; * *p* < 0.05, ** *p* < 0.01, *** *p* < 0.001, **** *p* < 0.0001.

For allergic sensitization, mice were intraperitoneally immunized on days 0 and 14 with 10 µg OVA (grade V; Sigma-Aldrich, St. Louis, MO, USA) in phosphate-buffered saline (PBS) and 67% (*v*/*v*) alum (Alu-Gel-S Suspension; Serva Electrophoresis, Heidelberg, Germany) in a total volume of 150 µL (PBS/OVA/OVA group) or with PBS and alum alone (PBS/PBS/PBS group). Subsequently, mice were challenged intranasally with 100 µg OVA in PBS in a total volume of 30 µL (PBS/OVA/OVA group) or 30 µL PBS alone (PBS/PBS/PBS group) on days 21 to 23 (Figure 1A). Before each challenge, mice were anesthetized with 5% (*v*/*v*) isoflurane (Isocare; Inhalation vapor, Animalcare Ltd., York, United Kingdom) at an airflow rate of 3 L/min in a UniVet Porta anesthesia machine (Groppler Medizintechnik, Deggendorf, Germany). 24 h after the last challenge, airway hyperresponsiveness (AHR) was assessed with whole-body plethysmography (Buxco Electronics Inc., DSI, New Brighton, MN, USA). Mice were conscious and unrestrained while they were exposed to 0, 12.5, and 25 mg/mL of aerosolized methacholine (acetyl-β-methyl-choline chloride; Sigma-Aldrich) in PBS. AHR was expressed by the dimensionless parameter enhanced pause (PenH) as described elsewhere [16]. Mice were euthanized and organs were harvested on day 25.

### 2.3. Allergen-Specific Tolerance Induction

Mice were tolerized intragastrically five times with 1 mg of OVA (grade V; Sigma-Aldrich) in a total volume of 300 µL buffer (0.2 M NaHCO_3_ with 1% (*w*/*v*) glucose in PBS, pH = 8.5) (OVA/OVA/OVA group) on days −11 to −7 followed by sensitization on days 0 and 14 and challenge on days 23 to 25 (Figure 1A). PBS/OVA/OVA and PBS/PBS/PBS mice received bi-carbonate buffer on days −11 to −7 instead.

### 2.4. Differential Cell counts in Bronchoalveolar Lavage Fluid (BALF)

Lungs were lavaged with 1 mL ice-cold PBS. BALF was centrifuged (300× *g* for 5 min at 4 °C). Pelleted cells were resuspended in PBS and 4 × 10^4^ cells were spun onto microscope slides (800× g for 3 min; Shandon Cytospin, Shandon Southern Instruments, Thermo Fisher Scientific, Waltham, MA, USA), air-dried and stained with hematoxylin and eosin (H&E; Hemacolor^®^, Merck KGaA, Darmstadt, Germany). On average, a total of 220 cells (macrophages, eosinophils, lymphocytes, and neutrophils) per slide were counted under a light microscope (×100 magnification; Nikon Eclipse, Nikon, Minato, Tokyo, Japan).

### 2.5. Lung Cell Isolation and in Vitro Stimulation

Lungs of terminally anesthetized mice were excised and processed as described elsewhere [17]. Briefly, lungs were minced and digested in 6 mL RPMI-1640 media (Gibco^®^, Thermo Fisher Scientific, Waltham, MA, USA) containing 0.05 mg/mL Liberase TL (Roche, Basel, Switzerland) and 0.5 mg/mL DNAse (Sigma-Aldrich) for 45 min at 37 °C in 5% CO_2_ atmosphere. Next, the digested tissue was forced through a 70 µm cell strainer and erythrocytes were lysed in 3 mL ammonium-chloride-potassium (ACK) Lysing Buffer (BioWhittaker^®^, Lonza Group Ltd., Basel, Switzerland) for 2 min. Lung cells were resuspended (5 × 10^6^ cells/mL) in RPMI-1640 containing 10% fetal calf serum (FCS), 2 mM mercaptoethanol, 2 mM L-glutamine and 100 µg/mL gentamycin (Sigma-Aldrich). 100 µL cell suspensions were plated into 96-well plates and incubated either with complete RPMI or with 100 µg/mL endotoxin-free OVA (Endo-Grade; Hyglos) in complete RPMI for 72 h at 37 °C in 5% CO_2_ atmosphere. After incubation, supernatants were collected and analyzed for the production of cytokines (IL-4, IL-5, IL-10, IL-13, and IFNγ) with commercially available ELISA kits following the manufacturer’s instructions (Ready-SET-Go!™ Kit, eBioScience™, Thermo Fisher Scientific).

### 2.6. Lung Histology

Lungs were infiltrated with 7.5% (*v*/*v*) formaldehyde for histology. Formalin-fixed lungs were transferred into 70% ethanol and subsequently embedded in paraffin. Sections (5 µm) were stained either with H&E or with Periodic Acid-Schiff (PAS; Sigma-Aldrich). The histological pathology score was evaluated according to Zaiss et al. [18] with modifications. Stained sections were scored according to following criteria regarding: (i.) perivascular and peribronchiolar inflammation (H&E) (0 = no inflammation; 1 = single scattered leukocytes; 2 = aggregates less than 10 cells thick; 3 = aggregates more than 10 cells thick; 4 = numerous coalescing aggregates more than 10 cells thick), (ii.) number of leukocytes in alveolar spaces (H&E) in 400× high power field (HPF) (0 = not present; 1 = 2–4 cells; 2 = 5–10 cells; 3 = >10 cells), and (iii.) number of PAS-positive cells per 50 airway epithelial cells in 5 randomly selected regions (0 = no cells; 1 = 1–12 positive cells; 2 = 13–25 positive cells; 3 = >25 positive cells).

### 2.7. Collection of Serum 

On days −11 and 25, approximately 100 µL blood were collected by puncturing the facial vein. Serum was obtained by centrifuging the blood in microtainer^®^ SST™ tubes (BD Pharmingen™, Franklin Lakes, NJ, USA) at 15,000 g for 5 min. Serum was stored at −20 °C until analysis.

### 2.8. Detection of OVA-Specific Antibodies

Microtiter plates were coated with OVA (5 µg/mL; grade V) overnight, blocked with 1% (*w*/*v*) bovine serum albumin (BSA) and 0.05% (*v*/*v*) Tween in PBS for 6 h and subsequently incubated with serum samples collected on days −11 and 25 at 4 °C overnight. Sera were diluted 1:500 for IgG2a measurement. The next day, plates were washed and incubated with rat-anti mouse IgG2a (1:500, BD Pharmingen™) at 4 °C overnight. On the next day, plates were washed and incubated with horseradish peroxidase-conjugated mouse anti-rat IgG (1:2000; Jackson ImmunoResearch Europe Ltd., Ely, United Kingdom) at 37 °C for 1 h followed by incubation at 4 °C for 1 h. Plates were washed again and 1 mM ABTS (Sigma-Aldrich) in 70 mM citrate-phosphate buffer (pH = 4.2; Sigma-Aldrich) was added for colorimetric measurement. Absorption was measured at 405 nm with a SparkControl Magellan plate reader (Tecan, Männedorf, Switzerland).

### 2.9. Rat Basophil Leukemia (RBL) Cell-Based Assay

RBL cell mediator release assay was performed as described elsewhere [19]. Briefly, RBL 2H-3 (ATCC, Manassas, VA, USA) cells were plated into 96-well plates (4 × 10^4^ cells/well) and incubated with serum samples (1:300) from days −11 and 25 for 2 h at 37 °C in a 5% CO_2_ atmosphere. Cells were washed with Tyrode’s buffer (137 mM NaCl, 5.6 mM d-glucose, 2.7 mM KCl, 1.8 mM CaCl_2_, 1.1 mM MgCl_2_, 0.4 mM NaH_2_PO_4_, 12 mM NaHCO_3_, 10 mM 4-(2-hydroxyethyl)-1-piperazineethanesulfonic acid (HEPES) and 0.1% (*w*/*v*) BSA; pH = 7.4; Sigma-Aldrich) and degranulation of cells was induced by incubation with 0.3 µg/mL OVA in Tyrode’s buffer. Supernatants were analyzed for β-hexosaminidase content by incubation with 80 μM 4-methylumbelliferyl-*N*-acetyl-β-d-glucosaminide (Sigma-Aldrich) and measuring fluorescence at λ_ex_: 360 nm/λ_em_: 465 nm with a SparkControl Magellan plate reader. Results show the percentage of total β-hexosaminidase release in serum samples compared to positive controls after adding 1% (*v*/*v*) Triton X-100 (Sigma-Aldrich) in double-distilled water.

### 2.10. Human Serum Samples

Permission to test human serum samples, which had been collected for a previously published study [20], was granted by the ethics committee of the Medical University of Vienna (EK Nr. 1734/2019). Sera of 20 allergic patients, 20 SIT-treated patients, and 20 healthy controls (age 20–58) were evaluated. Samples were gender-matched (females: *n* = 10 in allergic and healthy group; *n* = 11 in the SIT-treated group). Allergic patients were sensitized against grass pollen, birch, hazelnut, house dust mite, herb pollen, mugwort, cat epithelium, and mold. Single patients reported allergies against cockroach, bee, and wasp venom as well as plantain. The majority of allergic (*n* = 12) and SIT-treated patients (*n* = 12) had a known history of poly-sensitization. SIT-treated patients were often treated against more than one allergen (*n* = 9). SIT-treated patients were most commonly treated against allergens derived from bee and wasp venom (*n* = 8), house dust mite (*n* = 5), grass (*n* = 6), and birch pollen (*n* = 5).

### 2.11. Measurements of Serum Samples by FTIR Spectroscopy

Serum samples from sham-treated, allergic, and tolerized mice collected on day −11 and on day 25 (*n* = 60; 30 samples per experiment; see Section 2.7), as well as serum samples from healthy, allergic, and SIT-treated patients (*n* = 60; see Section 2.10), were subjected to FTIR analysis. 8 µL of serum samples were transferred onto a 384-well microtiter IR light transparent silicon plate (Bruker Optics GmbH, Ettlingen, Germany) and left for drying at 30 °C for 30 min to create a thin and transparent film. The FTIR measurements were carried out as described previously [12] using an HTS-XT microplate adapter coupled to a Tensor 27 FTIR spectrometer (Bruker Optics GmbH) Spectra acquisition was performed in transmission mode in the spectral range of 4000 to 500 cm^−1^, using the following parameters: 6 cm^−1^ spectral resolution, zero-filling factor 4, Blackmann-Harris 3-term apodization and 32 interferograms were averaged with background subtraction for each spectrum. The sample spectra were collected after background spectra were taken against an empty cell on the plate. At least 20 independent measurements were obtained per mouse and human sera samples and subjected to data analysis as described below.

### 2.12. Spectral Data Quality Assessment

To evaluate the spectral data quality of FTIR measurements, the following data analyses were performed. First, the obtained spectra were analyzed using the quality test tool of the OPUS software (version 7.8.5; Bruker Optics) to assess the quality of spectra with respect to absorbance values, signal-to-noise ratio and intensity of the water vapor lines. This analysis was followed by performing the Hotelling’s T^2^ versus *Q* residuals charts, using the mean-centered data to determine the outliers. Above 95% of the spectra passed the quality test evaluation. The outlying spectra, which were far from the origin of the Hotelling’s T^2^ versus *Q* residuals charts’ origin, were excluded from further data analysis.

### 2.13. Spectral Data Pre-Processing

FTIR spectral data pre-processing was comprised of the following steps: (i.) truncating the spectral wavenumbers to the spectral region (1800–900 cm^−1^), (ii.) calculation of polynomial order of second derivatives using the Savitzky–Golay algorithm with 15 smoothing points of windows sizes, (iii.) multiplicative scatter correction (MSC) to account for offset and baseline effects, (iv.) unit vector normalization to correct different scales of the spectra, which might have arisen from different thickness of the samples on the IR plate, and (v.) scaling the spectra before unsupervised or supervised classifications.

### 2.14. Unsupervised Learning: Principal Component Analysis (PCA)

Principal component analysis (PCA) is usually performed for data reduction to deal with high dimensional collinear data sets, such as FTIR spectral data, to analyze the main variations pattern. Since mouse samples from two independent experiments were included in the analysis, Piecewise Direct Standardization (PDS) was used for data standardization. Pre-processing, visualization, and unsupervised classification (PCA) of the FTIR spectra were performed by using The Unscrambler X (version 10.5) software and Python programming language with Scikit-learn library.

### 2.15. Supervised Learning: Deep Learning

FTIR spectral data were subjected to supervised machine learning using MATLAB (Release 2018b, The MathWorks Inc., Natick, MA, USA) combined with the Deep Learning Toolbox, running on a quad-core CPU laptop using single NVIDIA GeForce GTX 1060 (1152 CUDA cores, Santa Clara, California, USA) graphics card. The Deep Learning Toolbox from MATLAB R2018b was applied to construct a convolutional neural network learning model. The pre-processed spectral data were split into the three subsets, required for the supervised machine learning, using the following ratios: 65% of the spectra were assigned to the training set, 20% of the spectra to the validation set, and 15% of the spectra to the test set. To increase the robustness 10-fold cross-validation was implemented. The set for training the network comprised input and target vectors of the assigned set by computing the gradient and updating the network weights and biases. The validation set was used to validate that network by generalizing and allowing stopping training before overfitting by monitoring the error on the validation set during the training process. To assess the suitability of the model on a plausible real-world scenario, an independent test set was applied to analyze the network accuracy on data not being used in training or validation. As for the classification merits of the model, the following terms were calculated: (i.) True Positive (TP), which corresponds to the number of observations that are originally positive and also classified as positive; (ii.) True Negative (TN), which corresponds to the number of observations that are originally negative and also classified as negative; (iii.) False Positive (FP), which corresponds to the number of observations that are originally negative but classified as positive, and (iv.) False Negative (FN), which corresponds to the number of observations that are originally positive but classified as negative. The specificity, which corresponds to the true negative rate results of each predicted class, was based on the TN and FP. The sensitivity (also known as recall), which corresponds to the true positive rate (TPR) results, was calculated by TP and FN numbers for the considered class. The positive predicted values (PPV), referred to as precision, were calculated by TP and FP values. For calculation of the performance metrics, the pooled data of the test set were displayed in one matrix. Chord diagrams as plots of confusion matrices were prepared to show the misclassification of the supervised model based on the test set. 

### 2.16. Convolutional Neural Networks (CNN) Model Architecture

Due to the one-dimensional nature of the FTIR transmission spectral data set, which comprises a multi-variable matrix shape (spectral wavenumbers features are ordered in columns with different samples in rows), the one-dimensional (1D) CNN model was used. CNNs are feed-forward neuronal networks that contain neurons with learnable weights and biases. In our model, the specific variables for the model were optimized by using Bayesian optimization. These variables were used for both options of the training algorithm and parameters of the network architecture. This optimization process allowed minimizing the classification error on the validation set and later on by loading the best network from disk and evaluating it on the test set. The variables optimized in the model were: (i.) network section depth that controls the depth of the network identical to convolutional layers, (ii.) initial learning rate, (iii.) momentum that leads to lesser reduction noise inherent to stochastic gradient descent, and (iv.) L2 regularization strength to help to prevent overfitting by looking at the optimal space for regularization strength to find a better value for the model. The convolutional layers were also created by optimization of the number of filter sizes and the number of filters in convolutional layers. The pre-processed FTIR spectra were used as input, followed by the convolutional layers with features detectors at optimized kernel sizes and polling layers to extract the important spectral features with a rectified linear unit (ReLU) as the active function were applied. This procedure allowed the learning model to include more complex features. The pooling layers were running through data by sliding a defined window of height. Two additional optimized convolutional layers with max pooling layers were added to the model to learn more complex features and to prevent overfitting. Subsequently, a fully connected layer was added with the softmax activation function to monitor the probability of the classification of the output classes. Thus, the final output layer had probability values between 0 and 1.

### 2.17. Statistics

The FTIR spectral data were evaluated using a normality test to decide whether the parametric or nonparametric statistical test should be used. For this purpose, the Mardia and Royston tests were applied [21]. These resulted in a non-normality assumption (the degree of significance, *p* < 0.0001), considered as monotonic but also nonlinear distribution between the variables of the spectral features. The one-way analysis of variance and Dunnett’s multiple comparison tests (GraphPad Prism 6; GraphPad Software, Inc., San Diego, CA, USA), which were considered statistically significant at *p* < 0.05, were used to analyze each different treatment group of mice. Immunological parameters of mice were compared by performing a two-way analysis of variance, followed by Tukey’s Multiple Comparison Test, except for the evaluation of histopathology, where one-way analysis of variance, followed by Tukey’s Multiple Comparison Test was applied using GraphPad Prism 7 (GraphPad Software Inc.). All data are shown as mean ± SEM. Significant differences were considered at *p* < 0.05 (*), *p* < 0.01 (**), *p* < 0.001 (***), and *p* < 0.0001 (****).

## 3. Results

### 3.1. Allergen-Specific Oral Tolerization Reduces AHR to Methacholine and Suppresses Recruitment of Eosinophils to the Lung

FTIR spectroscopy was employed to investigate the impact of experimental allergy or allergen-specific oral tolerization on biomolecules, such as lipids, proteins, and carbohydrates, in sera from mice. To obtain the respective sera, allergic airway inflammation was induced in BALB/c mice according to a published protocol [22] with minor adaptations. For induction of specific tolerance, mice were pre-treated intragastrically with 1 mg of OVA in a bi-carbonate buffer (tolerized group; OVA/OVA/OVA) or bi-carbonate buffer only (PBS/OVA/OVA) on days −11 to −7. As expected, intragastric treatment with 1 mg OVA (Figure 1A) reduced AHR induced by 25 mg/mL methacholine and measured by whole-body plethysmography in OVA/OVA/OVA mice compared to sensitized and challenged controls (PBS/OVA/OVA) (Figure 1B). There was no difference between OVA/OVA/OVA mice and sham controls (PBS/PBS/PBS; Figure 1B).

In parallel, bronchoalveolar lavage cytospins showed that intragastric OVA reduced eosinophil recruitment to the lung in comparison to PBS/OVA/OVA mice (Figure 1C,D) and these levels were similar to PBS/PBS/PBS mice. Additionally, recruitment of inflammatory cells to perivascular and peribronchiolar alveolar space, mucus production and goblet cell hyperplasia were suppressed in OVA/OVA/OVA mice compared to PBS/OVA/OVA mice, leading to a histopathological score similar to the score observed in PBS/PBS/PBS mice (Figure 1F).

### 3.2. Allergen-Specific Oral Tolerization Reduces Th2 Cellular and Humoral Responses

**Figure 2:**
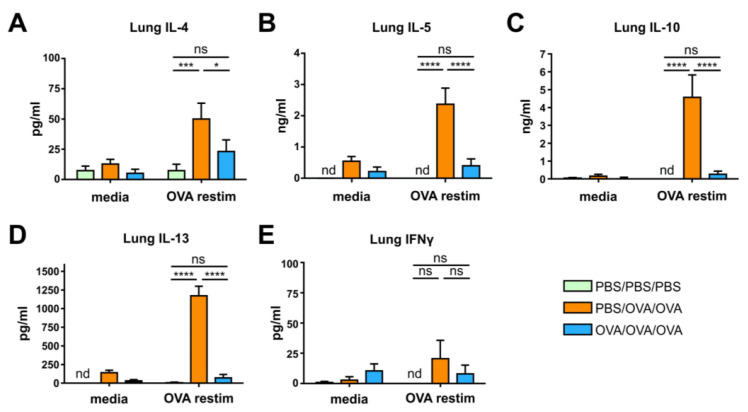
Prophylactic oral treatment with ovalbumin (OVA) reduces the development of cellular Th2 responses in the lung. (**A**–**E**), Levels of IL-4 (**A**), IL-5 (**B**), IL-10 (**C**), IL-13 (**D**), and IFNγ (**E**) after medium- and OVA restimulation of lung cells from mice treated as in Figure 1A. Graphs show pooled results from 2 independent experiments with 9–10 mice per group. PBS, phosphate-buffered saline. Error bars show mean ± SEM. nd not detectable; ns not significant; * *p* < 0.05, *** *p* < 0.001, **** *p* < 0.0001.

At sacrifice, lungs were collected and single cells were incubated with media or OVA. Upon intragastric OVA-application, levels of IL-4, IL-5, IL-13, and IL-10 were reduced in comparison to PBS/OVA/OVA mice as measured in supernatants by ELISA with comparable levels measured in PBS/PBS/PBS mice (Figure 2A–D).

**Figure 3:**
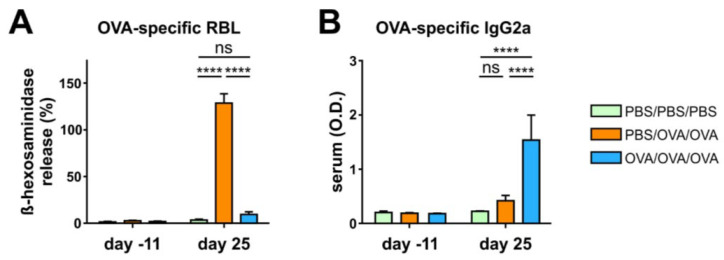
Prophylactic oral treatment with ovalbumin (OVA) leads to reduced allergen-specific IgE and increased allergen-specific IgG2a in serum. (**A**), OVA-specific IgE-mediated release of β-hexosaminidase via mast cell degranulation. (**B**), Levels of OVA-specific antibodies IgG2a in serum collected at the beginning and the end of the experiment. Graphs show pooled results from 2 independent experiments with 9–10 mice per group. O.D., optical density; PBS, phosphate-buffered saline. Error bars show mean ± SEM. ns not significant; **** *p* < 0.0001.

The levels of IFNγ did not differ among the groups (Figure 2E). In serum, the OVA-specific release of β-hexosaminidase in RBL cells was reduced in OVA/OVA/OVA mice compared to PBS/OVA/OVA mice and did not differ compared to PBS/PBS/PBS mice (Figure 3A). Intragastric tolerance-induction increased levels of allergen-specific IgG2a in the serum of OVA/OVA/OVA mice compared to PBS/PBS/PBS and PBS/OVA/OVA mice (Figure 3B).

### 3.3. FTIR Spectroscopy Combined with Unsupervised Machine Learning of Serum Samples Stratifies Healthy, Allergic, and Tolerized Mice 

**Figure 4:**
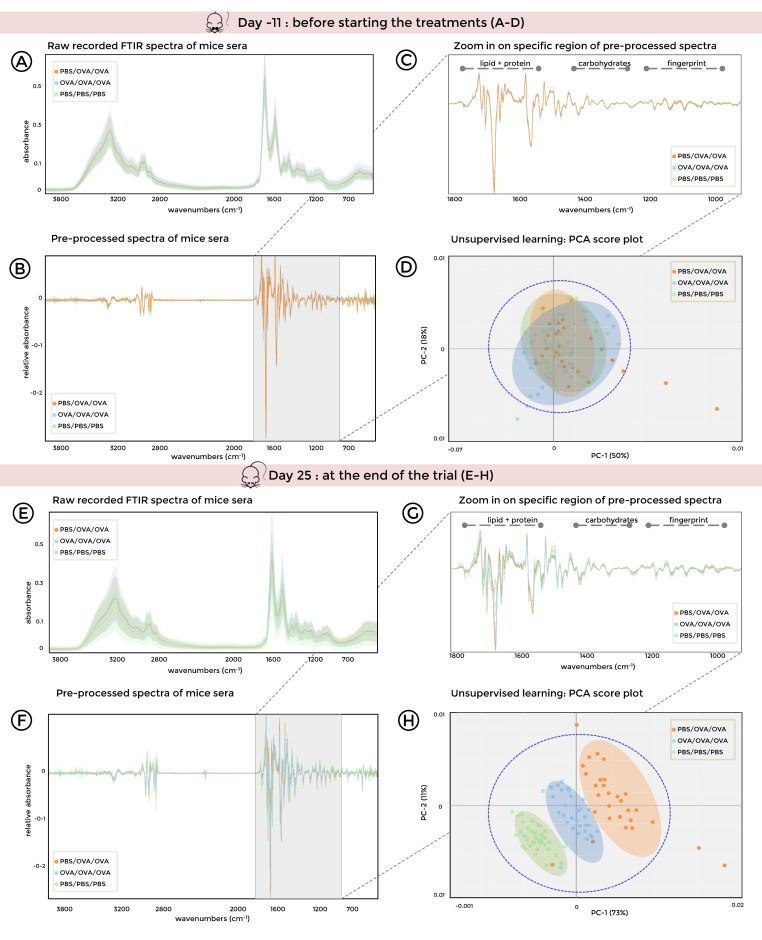
Principal component analysis (PCA) of Fourier-Transform Infrared (FTIR) spectra from sera stratifies healthy, allergic, and tolerized mice. (**A**–**D**), Mouse sera were collected on –11 prior to treatments and (**E**–**H**), on day 25 at the end of the trial. (green: PBS/PBS/PBS; orange: PBS/OVA/OVA; and blue: OVA/OVA/OVA). FTIR spectra are shown as (**A**,**E**), unprocessed, and (**B**,**F**), pre-processed with wavenumbers selected for comparison highlighted in grey. (**C**,**G**), significant biomolecule regions. (**D**,**H**), PCA score plots; principal components (PC-1 and PC-2) within 95% confidence ellipse. OVA, ovalbumin; PBS, phosphate-buffered saline.

Serum samples were collected before the start of the treatment on day −11 and at the sacrifice on day 25 and subsequently analyzed by FTIR spectroscopy (Figure 4A,E). As depicted in Figure 4B,F, the most discriminatory spectral characteristics in sera were found in the spectral range from 900 to 1800 cm^−1^. Thus, this spectral window, which includes the ester-linked (1710–1760 cm^−1^), the protein-(1500–1720 cm^−1^), the collagen-(1190–1350 cm^−1^), the phosphate diester-(1220–1250 cm^−1^), and the carbohydrate-related (950–1200 cm^−1^) range, was used for unsupervised machine learning assisted data analyses (Figure 4C,G).

Principal component analysis (PCA) revealed that spectra of serum samples taken before the start of the treatment were intermingled, generating one big cluster (Figure 4D: PC-1 50% and PC-2 18%), while spectra obtained from allergic and tolerized mice after treatment were separating from sham-treated mice, resulting in three distinct clusters alongside PC-1 (Figure 4H: 73% PC-1 and 11% PC-2 within the 95% confidence ellipses). These results show that FTIR spectroscopy coupled to machine learning is a suitable tool to discriminate healthy, allergic, and tolerized mice based on the metabolic fingerprints of their sera.

### 3.4. Stratification of Allergic, SIT-Treated Patients and Healthy Individuals was Enabled by FTIR Spectroscopy Combined with Deep Learning

**Figure 5:**
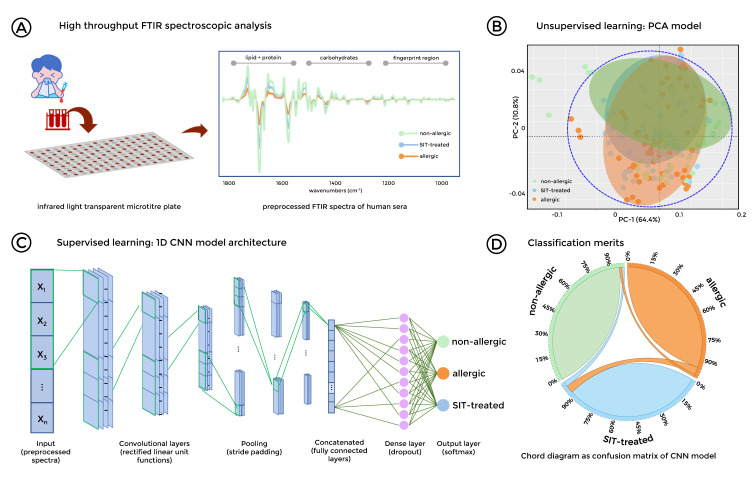
Machine learning empowered Fourier-Transform Infrared (FTIR) spectra from sera stratifies healthy, allergic, and SIT-treated subjects. (**A**), Succinct workflow for spectroscopic measurements with pre-processed FTIR spectra. Color codes: green: non-allergic donors; orange: allergic patients; blue: SIT-treated patients. (**B**), Unsupervised learning: Principal component analysis (PCA) score plots showing PC-1 and PC-2 within 95% confidence ellipse. (**C**), Supervised learning: Convolutional Neural Network (CNN) model for classification of FTIR spectra of human sera from non-allergic, allergic, and SIT-treated patients. It incorporates convolutional layers for spectral feature extraction and fully connected layers with dropout operations employed in dense layers as an output layer of size 3 for classification of respected groups, which is activated by softmax function. (**D**), classification merits: confusion matrix results based on the pooled test data represented as a chord diagram to display the inter-relationships within different groups.

To further explore the potential of FTIR spectroscopy for the field of diagnosis and clinical therapeutic monitoring of allergic patients, a pilot study was conducted using sera from healthy, allergic, and SIT-treated patients (*n* = 20 per group) (Figure 5A). Human serum samples were subjected to FTIR spectroscopy, spectral data processing, and data analyses as described for the mouse samples. PCA analysis revealed two clusters, one for the non-allergic subjects and one for both allergic and SIT patients (Figure 5B). However, due to the nature of the human data set, which is more heterogonous compared to genetically identical mouse samples, some of the samples were intermingled in each cluster (Figure 5B). Furthermore, since infrared spectroscopic data provide information on all components of a sample in one spectrum, the correlation of co-varying features of different components represents a major challenge. This is particularly the case for biological systems in which samples have multiple cellular biochemical components, such as proteins, lipids, carbohydrates, and species-specific cellular wall structures in the fingerprint region of the spectra. Thus, multiclass classification deep learning with the CNN model, which is one of the emerging supervised machine learning methods [23], was utilized to analyze the FTIR spectra from human serum (Figure 5C). The misclassified portion of SIT-treated and allergic patient samples were slightly higher than non-allergic donors (Figure 5D and Figure S1). The overall accuracy was 93.9% for the 10-fold cross-validation results of the test data (Figure S2). The specificity values for spectral discrimination of allergic and SIT-treatment groups were 96% whilst the non-allergic donor specificity was 97%. The established deep learning classification approach through interrogated FTIR spectra successfully discriminated serum samples from allergic, SIT-treated and healthy human patients with true positive rates of 93.3%, 91.7%, and 96.7% and positive predictive values of 93.3%, 93.2%, and 95.1%, respectively (Figure 5D and Figure S1). The accuracy changes over epochs as well as the effects of loss of training data are shown in Figure S3. The detailed topology of the proposed CNN model is displayed in Figure S4.

## 4. Discussion

The urgent need to develop novel methods for rapid and inexpensive diagnosis of allergies is reflected by the global increase of allergic patients [24,25]. Thus, with our work we aimed to explore the potential of machine learning assisted FTIR spectroscopy as a potential clinical diagnostic and monitoring tool for allergies.

Both, allergic diseases and successful SIT are associated with drastic changes in cellular and molecular immune responses [26,27,28,29,30,31]. We previously showed that SIT-treated patients exhibited higher baseline levels of IL-10, a higher percentage of FoxP3+ regulatory T cells, and elevated levels of highly suppressive effector regulatory T cells in peripheral blood mononuclear cells compared to healthy controls, which agrees with previous findings in the literature [20,32,33,34]. Moreover, we showed that the numbers of FceRII-expressing B cells (CD19+/CD23+) were reduced in SIT-treated patients in comparison to allergic patients, reaching comparable levels to non-allergic controls [20]. Here, we show that levels of eosinophils and type 2 cytokines, such as IL-4, IL-5, and IL-13, in the lungs of allergic mice differ significantly from sham-treated mice and mice that were tolerized. Similarly, in serum typical Th2-associated allergen-specific IgE-mediated β-hexosaminidase release was increased in allergic mice compared to both sham-treated and tolerized mice. Interestingly, levels of allergen-specific Th1-associated isotype IgG2a were increased in tolerized mice in comparison to both allergic mice and sham controls. These results corroborate findings from our previous studies [19,35,36,37,38].

Besides altered levels of antibodies, cytokines, such as IL-4 and IL-5 [39,40], and chemokines, such as eotaxins [41,42] can be detected in serum and differ between healthy and allergic mice, as well as humans. Additionally, the levels of multiple metabolites in serum, such as inosine, l-tryptophan, or short-chain fatty acids are influenced by allergic diseases and their detection and quantification could have diagnostic potential in humans [43,44,45,46,47]. However, metabolomic profiling usually requires complex and expensive methods, such as chromatography-coupled mass spectrometry, which makes it unsuitable for routine clinical applications.

Experimental mouse models of allergic airway inflammation share many key immunological and pathological parameters with allergic asthma in humans. Accordingly, several mouse models have been established to investigate the pathological mechanisms of allergy as well as prophylactic or therapeutic approaches to treat or prevent allergy, both comparable and complementary to SIT in humans [31,43,48,49].

In human patients as well as in surrogate mouse models, allergic asthma leads to an influx of inflammatory cells and cytokines into the lungs and mucus hyperproduction [22,36,50,51,52,53,54]. Although the measurement of these parameters in BALF is simple, BALF sampling in humans requires local anesthesia, and complications, such as coughing, bleeding, and fever, have been reported [55,56,57]. Thus, alternative clinical diagnostics would be desirable. Notably, in COPD patients as well as in the corresponding mouse model, it was shown that matched BALF and plasma samples of mice and humans share more than half of their metabolites, suggesting that blood samples might be suitable and easily available surrogates for BALF to detect local changes in the lung during allergic airway inflammation. Importantly, this study suggested that results obtained from mouse models are suitable to predict the situation in human patients [58], prompting us to use experimental mouse models of OVA-induced airway inflammation and specific tolerance as a surrogate to assess the potential of FTIR spectroscopy for clinical diagnostics.

FTIR spectroscopy is a highly discriminatory method to obtain spectral fingerprints of complex samples based on their unique absorbance pattern [12,59]. Recently, the potential of FTIR spectroscopy for clinical diagnosis has been shown by stratification of plasma or serum samples of healthy controls and patients suffering from brain tumors, breast, or ovarian cancer [14,15,60,61]. In line with these results from cancer research, our current work revealed that FTIR spectroscopy is a suitable tool to stratify healthy, allergic, and tolerized mice based on serum samples. In combination with deep learning, FTIR spectroscopy also allowed the stratification of human serum samples derived from allergic and SIT-treated patients as well as healthy subjects. Human serum spectra collected by FTIR spectroscopy showed higher heterogeneity than mouse spectra, which might be in part caused by several confounding factors [62]. For instance, varying nutrition habits may be reflected in the metabolic patterns in serum, regardless of the immunological state of the individual [63,64,65]. Different diets as well as other environmental factors shape the composition of the gut microbiome, which in turn produces a broad variety of metabolites, such as short-chain fatty acids, flavones, indol- and phenyl derivatives, that are detectable in serum [43,66,67,68]. Furthermore, many patients with allergic asthma or allergic rhinitis use medication, such as corticosteroids or antihistamines, to reduce their symptoms [69,70]. It has been shown previously that drugs as well as metabolites thereof are detectable in blood shortly after administration, which might confound the results of metabolic analyses in serum [71,72]. Although the allergic and SIT-treated patients in our study suffered from different types of allergies and no information on dietary habits, gut microbial composition or intake of medication was available, we show that FTIR spectroscopy combined with deep learning methods stratifies allergic, SIT-treated, and healthy individuals, suggesting that it is a suitable tool to exploit universal allergic biomarkers and to eliminate confounding factors.

Detailed analysis of the absorbance spectra indicated specific compositional changes in serum that allowed the differentiation between healthy, allergic, and tolerized mice. The highest variability between the different experimental groups of mice was found in the lipid and protein regions of the spectra, probably reflecting the specific cytokine and antibody profiles of the differently treated mice. The treatment-specific FTIR spectral profiles in the lipid regions of the mouse sera (see Figure 4) are in line with results from nuclear magnetic resonance (NMR) and mass spectrometry [5,44], which revealed alterations in lipid metabolism in asthmatic patients, supporting our hypothesis that machine learning empowered FTIR spectroscopy could be an economic alternative for clinical diagnosis of allergies and for monitoring the outcome of SIT. Nevertheless, further investigations, including a larger human cohort, are required to identify and verify possible biomarkers in human sera. As this study was designed as a proof-of-principle investigation, we introduced FTIR spectroscopy as a diagnostic tool for samples from adult patients while a follow-up age-matched study could elucidate whether allergic diagnosis can be extended to children or elderly. Furthermore, as FTIR spectroscopy allows assessing multiple biochemical changes at once, it might also be suitable for monitoring of patients’ sera over a longer period of time, which would enable general practitioners to monitor and adapt the treatment strategy of SIT. In light of the growing need for patient-centered medicine, such an approach could present an important step towards a tailor-made therapy. Thus, a follow-up age-matched study should include a large cohort of patients receiving SIT with a broad variety of allergens to assess if FTIR spectroscopy is suitable to monitor the progress and outcome of SIT in the clinic.

## 5. Conclusions

In this proof-of-concept study, we present a novel, high-throughput strategy using FTIR spectroscopy for rapid and inexpensive diagnosis of allergy based on the analysis of serum samples. FTIR spectroscopy, combined with deep learning, allowed not only to differentiate between allergic and healthy patients but also stratified SIT-treated patients, suggesting that it might also be suitable for monitoring the efficacy of SIT in individual patients. However, it remains to be investigated, whether FTIR spectroscopic-based identification of the allergic status is limited to adults or can also be applied to samples collected from other cohorts, such as children.

## Supplementary Materials

The following are available online at https://www.mdpi.com/2218-273X/10/7/1058/s1, Figure S1: Heatmap confusion matrix with column and row summaries; plotted with 10-fold cross validation pooled data of test set, Figure S2: Box and whisker chart visualization for 10-fold cross validation spectral data analysis of the test set, Figure S3: Epoch vs loss graphs for representation classification accuracy of trained deep learning model, displaying a shaded background (on each minibatches) with the training data and the validation data set over training epochs, Figure S4: Representation of CNN model topology performed for spectral data analysis of human serum sample classification by displaying the meta parameters of the model attributes and initializers.
