# Supplementary material for: Machine Learning-Empowered FTIR Spectroscopy Serum Analysis Stratifies Healthy, Allergic, and SIT-Treated Mice and Humans
Source: Biomolecules. 2020 Jul 16;10(7):1058. doi: 10.3390/biom10071058 (PMC7408032; doi:10.3390/biom10071058)
Supplement: Supplementary file 1 [file biomolecules-10-01058-s001.pdf]

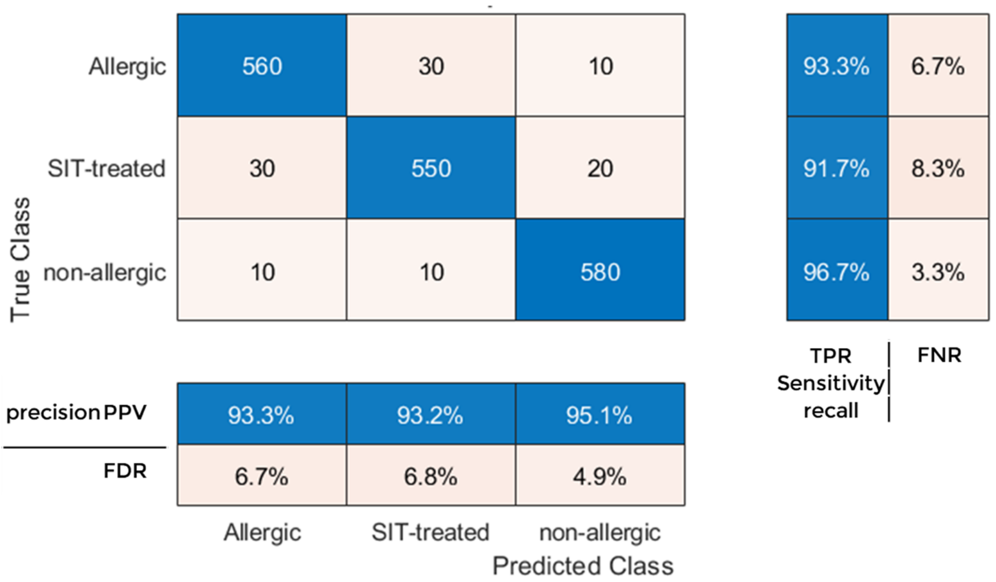

Figure S1. Heatmap confusion matrix with column and row summaries; plotted with 10-fold cross validation pooled data of test set. Diagonal within the confusion matrix shows the number of the spectra obtained correctly in the model within each group as being the true positive (TP) values. The columns summary located on the left side of the confusion matrix shows the number of correctly and incorrectly classified observations for each predicted class as percentages that are known as class-wise recall or true positive rate (TPR) or sensitivity values for each group. The row summary located on the bottom of the confusion matrix displays the number of correctly and incorrectly classified observations for each true class as percentages, which are sorted as class-wise precision or positive predictive value (PPV). False discovery rates (FDR) and false negative rates (FPR) values are shown as conceptualizing the error rates. The values of $TP_{allergic}$, $TP_{SIT\text{-}treated}$, and $TP_{non\text{-}allergic}$ are 560, 550, and 580, respectively which are represented in the diagonal. The values of false negative (FN) for each class (true class) are calculated by adding all errors in the row of that class. $FN_{allergic}$, $FN_{SIT\text{-}treatedd}$, and $FN_{non\text{-}allergic}$ are 40, 50 and 20, respectively. The values of false positive (FP) for each class (predicted class) are calculated by adding all errors in the column of that class. $FP_{allergic}$, $FP_{SIT\text{-}treated}$, and $FP_{non\text{-}allergic}$ are 40, 40 and 50, respectively. The value of true negative (TN) for each class (i.e. $TN_{allergic}$) is calculated by adding all columns and rows, excluding the row and column of class allergic; $TN_{allergic}$, $TN_{SIT\text{-}treated}$, and $TN_{non\text{-}allergic}$ are 1160, 1160 and 1170, respectively.

As for the calculation of the macro accuracy, the TP, TN, FP, and FN values are used in terms of.

$$Acc = \frac{TP + TN}{TP + TN + FP + FN}$$

resulting in 95.9%. Overall accuracy is calculated by summing the number of correctly classified values (TPs as in diagonals) and dividing it by the total number of values, resulting in 93.9%.

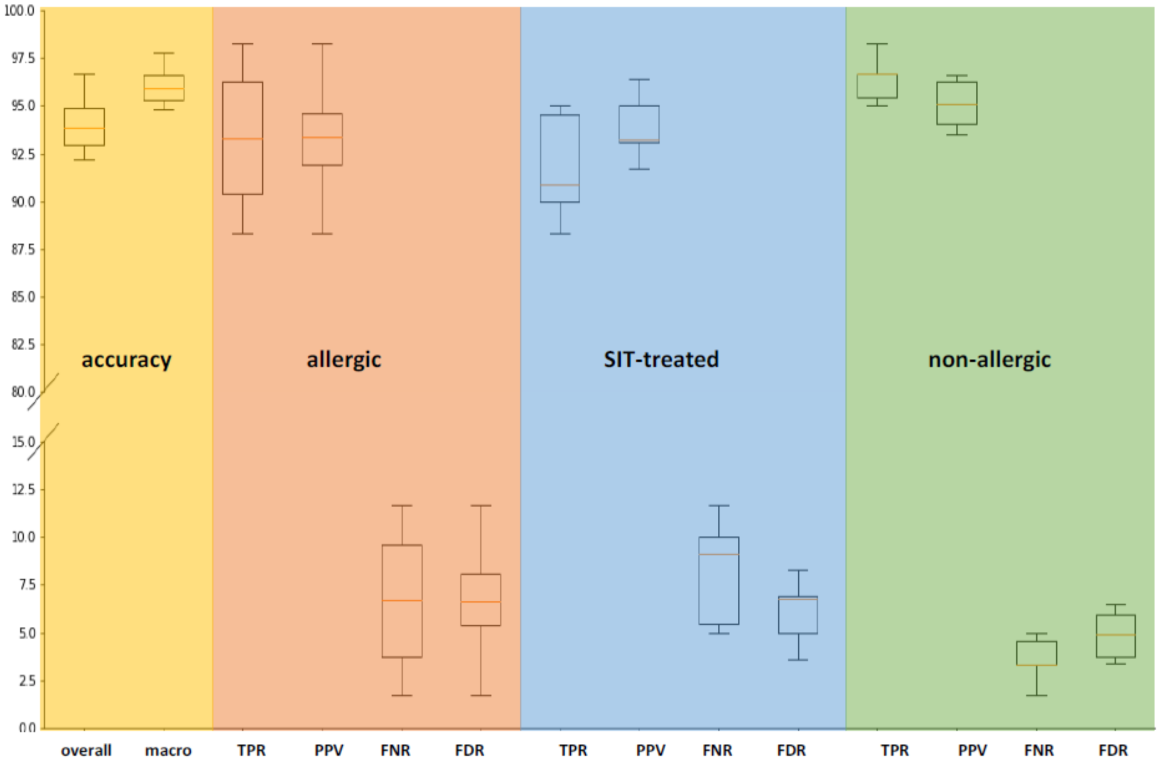

Figure S2. Box and whisker chart visualization for 10-fold cross validation spectral data analysis of the test set. x-axis shows classification merits of the deep learning model; from left to right: overall and macro accuracy, true productive rates (sensitivity, TPR), positive predictive values (precision, PPV), false negative rates (FNR), false discovery rates (FDR). Values for each group are indicated in colors: orange for allergic, blue for tolerized and green for non-allergic healthy donors, respectively. The y-axis represents percentages of the respected data shown on the x-axis.

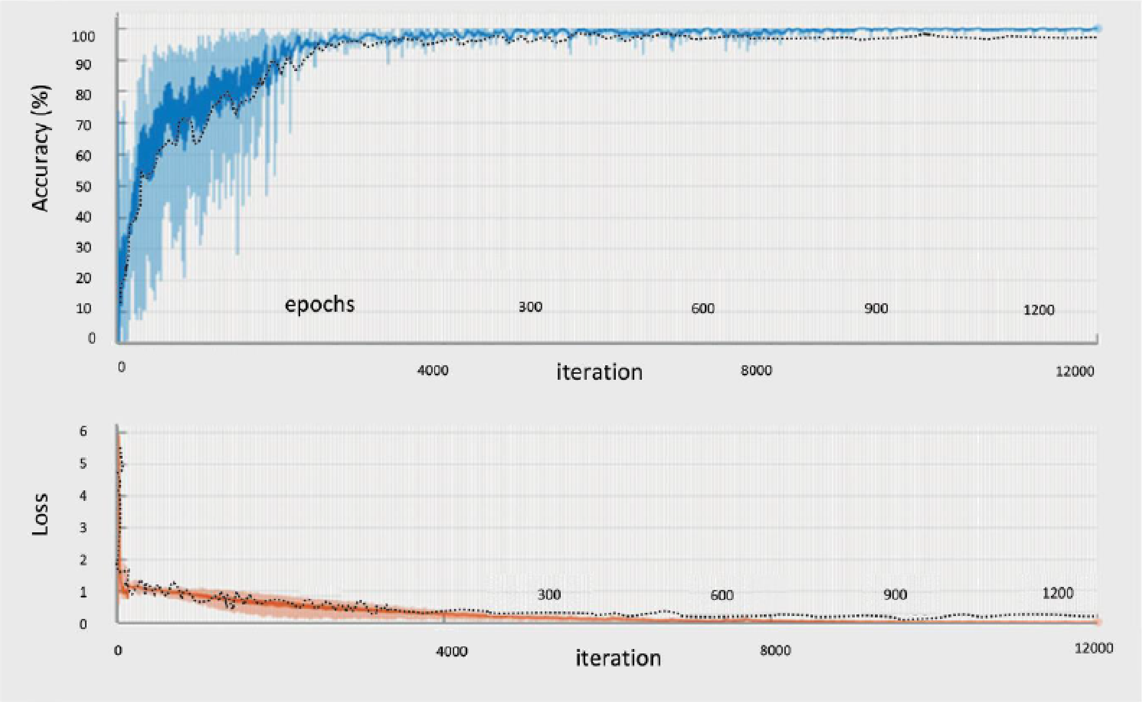

Figure S3. Epoch vs loss graphs for representation classification accuracy of trained deep learning model, displaying a shaded background (on each minibatches) with the training data and the validation data set over training epochs. **A)** Blue lines are representing the training data accuracy and validation set as black dots. **B)** Orange lines are representing the model loss based on cross entropy function on training set and validation set as black dots. The model has been trained as 12,000 iterations per 1200 epochs for the training set of 780 spectra using mini-batches (batch size: 64).

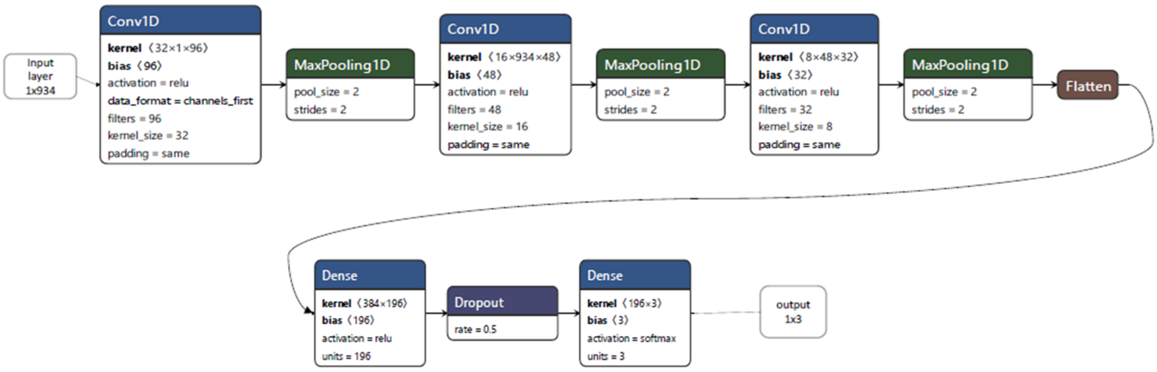

Figure S4. Representation of CNN model topology performed for spectral data analysis of human serum sample classification by displaying the meta parameters of the model attributes and initializers.
